# Investigating causal associations among gut microbiota, metabolites and autoimmune hypothyroidism: a univariable and multivariable Mendelian randomization study

**DOI:** 10.3389/fimmu.2023.1213159

**Published:** 2024-01-04

**Authors:** Xue Liu, Jie Yuan, Shuai Liu, Mulin Tang, Xue Meng, Xinhui Wang, Yuchen Li, Yuwei Chai, Chunjia Kou, Qingqing Yang, Juyi Li, Li Zhang, Qingbo Guan, Haiqing Zhang

**Affiliations:** ^1^Department of Endocrinology, Shandong Provincial Hospital, Shandong University, Jinan, Shandong, China; ^2^Department of Biostatistics, School of Public Health, Cheeloo College of Medicine, Shandong University, Jinan, Shandong, China; ^3^Key Laboratory of Endocrine Glucose & Lipids Metabolism and Brain Aging, Ministry of Education, Department of Endocrinology, Shandong Provincial Hospital Affiliated to Shandong First Medical University, Jinan, China; ^4^Department of Vascular Surgery, Shandong Provincial Hospital Affiliated to Shandong First Medical University, Jinan, Shandong, China; ^5^Shandong Clinical Medical Center of Endocrinology and Metabolism, Jinan, China; ^6^Institute of Endocrinology and Metabolism, Shandong Academy of Clinical Medicine, Jinan, China

**Keywords:** gut microbiota, gut metabolites, autoimmune hypothyroidism, mendelian randomization, thyroid-gut axis

## Abstract

**Background:**

Accumulating evidence suggests that the gut microbiota and its metabolites may be involved in autoimmune hypothyroidism. However, the causal association between gut microbiota, metabolites and autoimmune hypothyroidism remains to be determined.

**Methods:**

Instrumental variables were screened from the GWAS datasets of 211 gut microbiota taxonomic groups, gut microbiota-derived metabolites, and autoimmune hypothyroidism. Univariable Mendelian randomization (MR) and multivariable Mendelian randomization (MVMR) were used to analyse the potential causal relationship between autoimmune hypothyroidism, these metabolites, or these microbiota. During the MR analysis, we alternated multiple MR methods with different model assumptions to assess the consistency and robustness of the findings: inverse variance weighted (IVW), weighted median, MR pleiotropy residual sum and outlier (MRPRESSO) and MR−Egger methods. Reverse MR analysis was performed to assess the possibility of reverse causality. Finally, enrichment analyses were used to investigate potential biofunctions.

**Results:**

The IVW results of univariable MR showed that the phyla Actinobacteria, genus DefluviitaleaceaeUCG011, genus Eggerthella, family Defluviitaleaceae, genus Subdoligranulum, genus RuminococcaceaeUCG011, and genus Intestinimonas were associated with autoimmune hypothyroidism. After FDR adjustment, the absence of a causal relationship between gut microbiota and autoimmune hypothyroidism (*P_FDR_* > 0.05) suggested a possible marginal association. The results on gut metabolites showed that N-(3-furoyl)glycine, pipecolate, phenylalanine, allantoin, indololactate and alanine were associated with autoimmune hypothyroidism. After FDR correction, only indololactate was associated with hypothyroidism (OR=1.592; 95% CI, 1.228-2.065; *P_FDR_* = 0.036). Family Defluviitaleaceae and genus DefluviitaleaceaeUCG011 were suggestively significant in the MVMR. The results of reverse MR analysis showed no reverse causality between autoimmune hypothyroidism and the identified gut microbiota. Enrichment analysis revealed that several key regulatory pathways were significantly enriched.

**Conclusion:**

This study supported that there were beneficial or detrimental causal effects of gut microbiota and its metabolites on autoimmune hypothyroidism risk, which provides more theoretical support for mechanistic research on the “thyroid–gut” axis.

## 1 Introduction

As previous studies have indicated, the human gut microbiota is primarily composed of bacteria, with more than 90% comprising Firmicutes, Bacteroides, Actinomycetes, and Proteobacteria (1). The gut microbiota exerts a significant influence on the absorption of nutrients, regulation of epithelial development, guidance of innate immunity (1) and impact on adaptive immunity (2). In addition, gut microbiota ecology encompasses not only the gut microbiota but also a vast array of metabolic byproducts produced by the gut microbial community (3). The collective metabolic activities of the microbiota can be regarded as a virtual organ within the gut, relying on the human intestine, which plays a vital role in converting nutritional information from the gut into endocrine signals, influencing the surroundings, as well as remote organ metabolism (4), even being described as a “forgotten organ” (5). The gut microbiota could influence host immunity and physiology by producing metabolites (6). Studies have shown that metabolites produced by the gut microbiota, such as short-chain fatty acids, bile acids, and tryptophan metabolites, are the primary driving factors behind the influence of the gut microbiota on hosts (7). Furthermore, once breaching the intestinal barrier and entering the systemic circulation, the microbiota and its metabolites could promote the release of inflammatory factors, which may be one of the mechanisms involved in the activation of Hashimoto’s thyroiditis (HT) inflammation (8, 9).

Autoimmune hypothyroidism is one of the most prevalent thyroid disorders caused by HT, with a global prevalence rate of approximately 10–12% (10, 11). However, the aetiology of autoimmune hypothyroidism remains unclear. Numerous studies have provided evidence of the relationship between the gut microbiota and the thyroid (12). Since the first report of the association between gut microbiota and HT cases 40 years ago, a study had indicated that rats under pathogen-free conditions had a lower level of incidence of autoimmune thyroiditis compared to conventionally raised rats (13). Ishaq et al. illustrated the link between the modified composition and heightened diversity of the microbiota in individuals with HT compared to those in a healthy population, revealing an impaired microbiota in HT patients (14). Zhao et al. reported comparable findings, indicating a correlation between alterations in gut microbiota and thyroid function (15). With a foundation in genetic predisposition, the concept of the thyroid-gut axis (TGA) has emerged as a means to delve into the interconnectedness between the thyroid and the gut (16). While a correlation has been established between the gut microbiota, its metabolites, and hypothyroidism (17), the causal relationship remains unclear.

Due to ethical considerations and challenges in experimental procedures, it is challenging to establish causal relationships between them through experimental means. To achieve this, we utilized Mendelian randomization (MR) methods, which involve the application of instrumental variable (IV) analysis using genetic variants. The design is comparable to that of a randomized controlled trial. Given that there is usually a high degree of association between gut microbiota, we further verified it with multivariate Mendelian randomization (MVMR) after we used univariate MR to screen out meaningful microbiota.

In our study, we conducted a univariable and multivariable MR study using large-scale genome-wide association study (GWAS) datasets to explore the genetic relationship between the gut microbiota, its metabolites, and autoimmune hypothyroidism. This research yields fresh perspectives on the pathogenesis of thyroid disorders and offers potential clinical management strategies.

## 2 Materials and methods

### 2.1 Study design

In this study, univariable MR analysis and MVMR analysis were conducted on the overall profile of the gut microbiota, metabolites derived from the gut microbiota, and autoimmune hypothyroidism. MR analysis revealed a potential causal relationship between the gut microbiota and its metabolites and autoimmune hypothyroidism. Finally, reverse MR analysis was performed to assess the possibility of reverse causality.

### 2.2 Data sources

#### 2.2.1 Gut microbiota

The genetic data of the gut microbiota were derived from the most recent GWAS summary data provided by the MiBioGen Consortium (18). This large-scale GWAS involved 18,340 participants from 24 cohorts, representing multiple ethnicities. It encompassed a total of 211 taxonomic groups, including 131 genera, 35 families, 20 orders, 16 classes, and 9 phyla.

#### 2.2.2 Gut metabolites

The GWAS of the human metabolome from European populations (TwinsUK and KORA, N = 7824). The GWAS tested all 486 metabolite concentrations present in both datasets at each single-nucleotide polymorphism (SNP). We then obtained a list of 69 gut microbiota-derived metabolite signatures from all quantified metabolites in the GWAS via this URL (https://metacyc.org/).

#### 2.2.3 Autoimmune hypothyroidism

The genetic data regarding autoimmune hypothyroidism were collected from the publicly released DF8 data of the Finngen database, which was made available on December 1, 2022. In this dataset, hypothyroidism is defined as strict autoimmune hypothyroidism, comprising 36,321 cases and 250,926 controls. Detailed information regarding the Finngen database can be accessed at https://r8.risteys.finngen.fi/.

### 2.3 IV Selection

**Figure 1:**
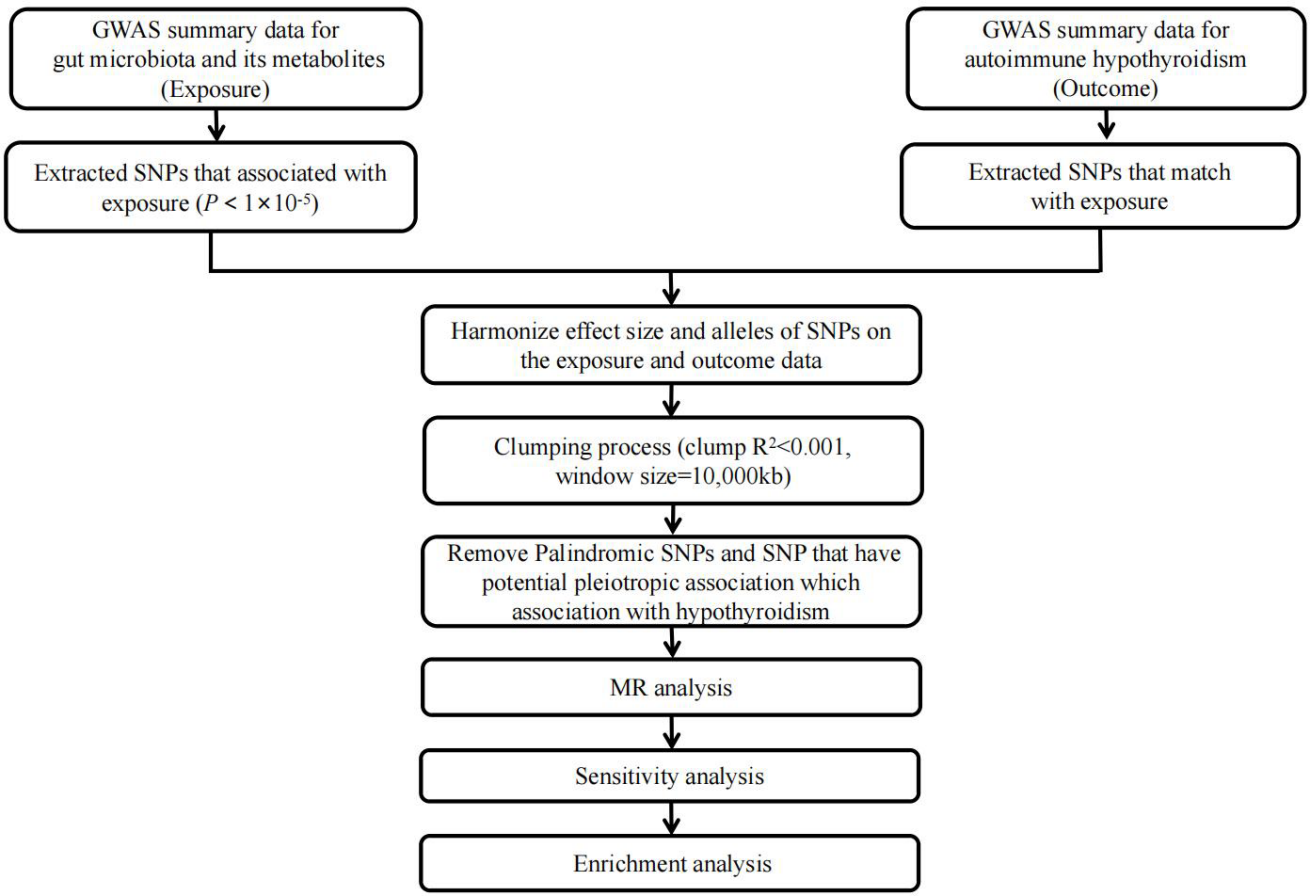
Study flow chart. The entire workflow of MR analysis. GWAS, Genome-wide association studies; MR, Mendelian Randomization.


**Figure 1** illustrates a concise study flow chart. In this study, the gut microbiota traits and gut microbiota-derived metabolites were considered as exposures, while autoimmune hypothyroidism was examined as the outcome. The IVs that met the assumptions of MR were strictly chosen. The specific screening steps were as follows: (1) The correlation between IVs and gut microbiota was evaluated using a threshold of *P*< 1 × 10^-5^. (2) Screening for independent SNPs (r^2^< 0.001, physical distance = 10000 kb). (3) Excluded the potentially pleiotropic SNPs that were associated with outcome at a *P* value less than 0.05 after Bonferroni correction. (4) To satisfy the strong association with exposure, SNPs with F statistic >10 were selected as IV.

$$\begin{array}{c} F_{i}=\frac{R_{i}^{2}\left(N_{i}-1-k\right)}{k-k\times R_{i}^{2}} \end{array};\;\;\;R_{i}^{2}=\frac{{\hat{\beta }}_{i}^{2}}{{\hat{\beta }}_{i}^{2}+{\left(se\left({\hat{\beta }}_{i}\right)\right)}^{2}N_{i}}$$

( $R^{2}$ means explained by genetic variation Exposure variance proportion of; β = effect; *N* = sample size; k = number of IVs). The *F*-statistic indicates the strength of the relationship between IVs and exposure (19). (5) Palindromic SNPs with intermediate allele frequencies were removed (20). (6) Checked in PhenoScanner (www.phenoscanner.medschl.cam.ac.uk) (21, 22), a platform with comprehensive information on the association of genotype and phenotype, to see whether these SNPs were associated with potential risk factors, such as other autoimmune diseases, iodine, selenium and other environmental factors.

### 2.4 Statistical analysis

In this study, univariable MR analysis was conducted to estimate the potential causal relationship between the gut microbiota, its metabolites, and autoimmune hypothyroidism. During the MR analysis, we alternated multiple MR methods with different model assumptions to assess the consistency and robustness of the findings. During the univariable MR analysis, we used the inverse variance weighted (IVW) method (23). As the main analysis method, the weighted median, MR Pleiotropy RESidual Sum and Outlier (MRPRESSO) and MR−Egger methods were used for the sensitivity analysis. At the same time, the MRPRESSO outlier test was used to test whether there were abnormal SNPs. To assess the heterogeneity among SNPs, Cochrane’s *Q* test was employed. The intercept of the MR−Egger regression test was used to estimate horizontal pleiotropy (24). Leave-one-out analysis was employed to examine whether the causal relationship between the gut microbiota, its metabolites, and autoimmune hypothyroidism was affected by individual SNPs (25). In MVMR analysis, we included the significant gut microbiota from the univariable analysis and tried to identify the independent gut microbiota. To explore whether autoimmune hypothyroidism had any causal effect on the identified important bacterial genera, we also performed a reverse MR analysis using SNPs associated with autoimmune hypothyroidism as IV (i.e., autoimmune hypothyroidism as exposure, identified pathogenic bacterial genera as a result).

To further explore the biological role of gut microbiota and metabolites in autoimmune hypothyroidism, we performed gene ontology (GO) and Kyoto Encyclopedia of Genes and Genomes (KEGG) enrichment analyses based on lead SNPs for all identified gut microbiota and metabolites. We mapped lead SNPs of causal microbiota and metabolites identified in autoimmune hypothyroidism to nearby genes.

All analyses in this study used the TwoSampleMR package (version 0.5.4) (26)MRPRESSO package (version 1.0) and MVMR package (version 0.3) in R software (version 4.1.1). Our results were corrected for multiple hypothesis testing using the false discovery rate (FDR), as the significance threshold was set at FDR-corrected *P* values < 0.05, while associations with *P *< 0.05 but not reaching the FDR-controlled threshold were reported as suggestive of association. Enrichment analysis was performed by the website tool “Metascape” (27). Reporting of the study follows the STROBE-MR statement (28) (**Supplementary Table 16**).

## 3 Results

### 3.1 IV selection

SNPs (rs1595463, rs16938435, rs6918730, rs11135366, rs2172426, rs4644504, rs2172426, rs11135366, rs17379710) were identified as potential pleiotropic outliers at the nominal significance level of 0.05 in the MRPRESSO outlier test. Detailed information can be found in the **Supplementary Tables**. The *F*-statistics, mostly above 10, suggested that the MR results were less likely to be affected by weak instrument bias. Furthermore, the MR−Egger regression analysis did not indicate any evidence of horizontal pleiotropy for the IVs (MR−Egger regression *P* > 0.05).

### 3.2 Univariable Mendelian randomization analysis

#### 3.2.1 Associations between the gut microbiota and autoimmune hypothyroidism

**Figure 2:**
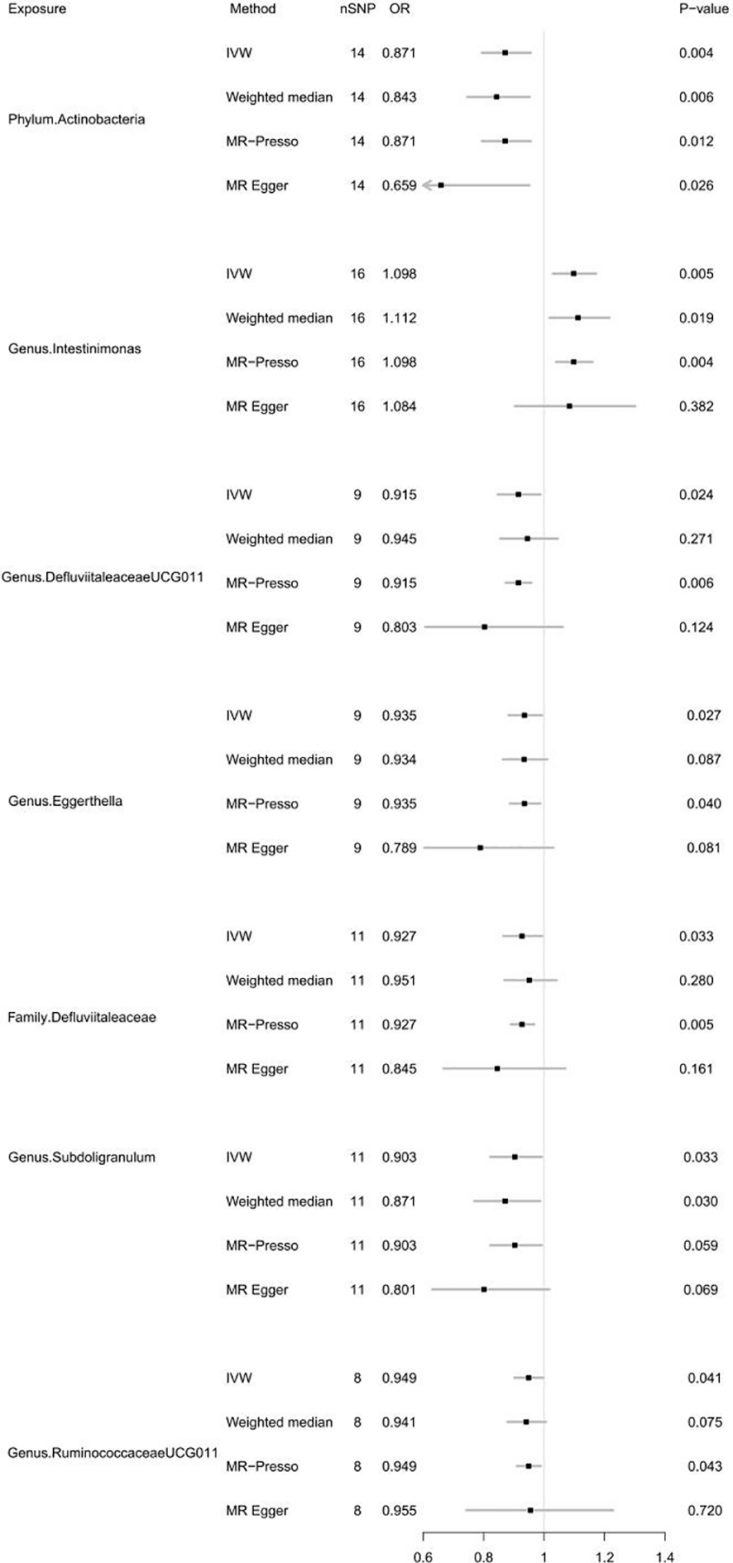
Forest plot of MR results for gut microbiota on autoimmune hypothyroidism. Method, statistical analysis methods; nSNP, number of SNPs; OR, odds ratio; P-value, significance *P*-value.

As shown in **Figure 2**, IVW analysis suggested that genetic prediction of the phylum Actinobacteria (OR=0.871; 95% CI, 0.794-0.956; *P* = 0.004), genus. DefluviitaleaceaeUCG011 (OR=0.915; 95% CI, 0.847-0.988; *P* = 0.024), Genus. Eggerthella (OR=0.935; 95% CI, 0.882-0.993; *P* = 0.027), Family. Defluviitaleaceae (OR=0.927; 95% CI, 0.865-0.994; *P* = 0.033), Genus. Subdoligranulum (OR=0.903; 95% CI 0.822-0.992; *P* = 0.033) and genus. RuminococcaceaeUCG011 (OR=0.949; 95% CI, 0.902-0.998; *P* = 0.041) was associated with a reduced risk of autoimmune hypothyroidism, but the genus. Intestinimonas (OR=1.098; 95% CI, 1.029-1.172; *P* = 0.005) was associated with an increased risk of autoimmune hypothyroidism. **Table 1** demonstrates that all *P* values in the heterogeneity tests were greater than 0.05, suggesting that our results were unlikely to be affected by heterogeneity bias. The MR−Egger intercept test suggested a minimal likelihood of horizontal pleiotropy (*P* > 0.05) (**Supplementary Table 17**). Furthermore, the leave-one-out analysis revealed that none of the potential outlier SNPs were included in the MR analysis (**Supplementary Material**).

**Table 1:** Results of heterogeneity and horizontal pleiotropy in the MR analysis of gut microbiota for autoimmune hypothyroidism.

Gut microbiota	Heterogeneity test			Horizontal pleiotropy		
	Method	Q	Q_pval	Egger_ intercept	se	pval
Phylum.Actinobacteria	MR Egger	13.104	0.362	0.018	0.011	0.125
	IVW	15.676	0.267			
Genus.Intestinimonas	MR Egger	10.515	0.724	0.001	0.008	0.884
	IVW	10.537	0.785			
Genus.DefluviitaleaceaeUCG011	MR Egger	2.033	0.957	0.014	0.014	0.344
	IVW	2.930	0.939			
Genus.Eggerthella	MR Egger	4.831	0.681	0.019	0.015	0.198
	IVW	6.487	0.593			
Family.Defluviitaleaceae	MR Egger	2.921	0.967	0.010	0.012	0.417
	IVW	3.581	0.964			
Genus.Subdoligranulum	MR Egger	9.919	0.357	0.010	0.009	0.284
	IVW	11.183	0.343			
Genus.RuminococcaceaeUCG011	MR Egger	4.817	0.567	-0.001	0.017	0.958
	IVW	4.820	0.682			

After FDR correction, the causal link between gut microbiota and autoimmune hypothyroidism was absent (*P_FDR_* > 0.05). In summary, phylum Actinobacteria, genus DefluviitaleaceaeUCG011, genus Eggerthella, family Defluviitaleaceae, genus Subdoligranulum and genus RuminococcaceaeUCG011 may have a borderline negative correlation with autoimmune hypothyroidism risk, and the genus Intestinimonas may have a borderline positive correlation with autoimmune hypothyroidism risk. For detailed FDR results, please refer to **Supplementary Table 8**.

#### 3.2.2 Associations between gut metabolites and autoimmune hypothyroidism

**Figure 3:**
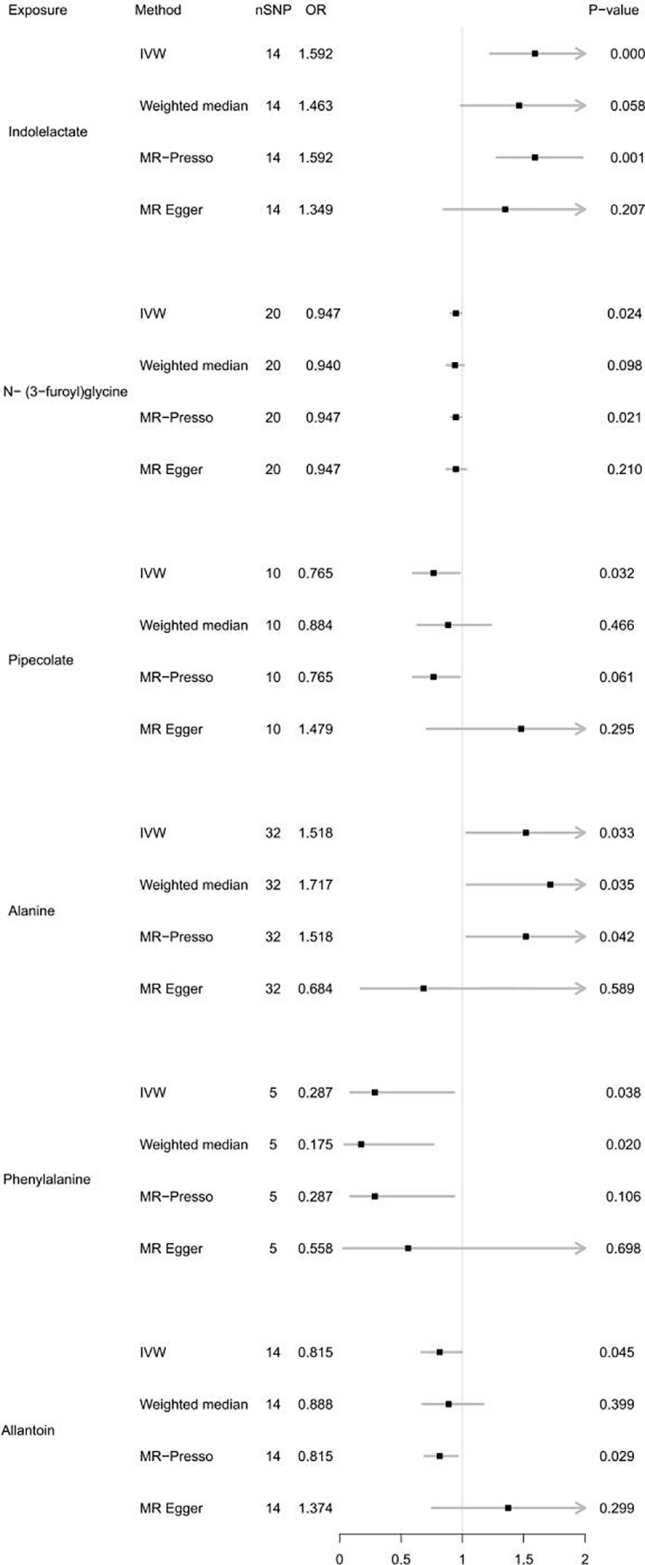
Forest plot of MR results for gut metabolites on autoimmune hypothyroidism. Method, statistical analysis methods; nSNP, number of SNPs; OR, odds ratio; P-value, significance *P*-value.

For the gut microbiota-derived metabolites included in the MR analysis, we observed suggestive estimated effects for 6 gut metabolites on autoimmune hypothyroidism. Our study revealed that gut metabolites, including indololactate and alanine, were associated with an increased risk of autoimmune hypothyroidism. The odds ratio (OR) values, estimated using the IVW method, were 1.592 (95% CI, 1.228-2.065; *P* = 4.55×10^-4^) and 1.518 (95% CI, 1.033-2.229; *P* = 0.033). N-(3-Furoyl)glycine, pipecolate, phenylalanine and allantoin were associated with a lower risk of autoimmune hypothyroidism, and their OR values by the IVW method were 0.947 (95% CI, 0.904-0.993; *P* =0.024), 0.765 (95% CI, 0.598-0.977; *P* =0.032), 0.287 (95% CI, 0.089-0.931; *P* =0.038), and 0.815 (95% CI, 0.667-0.996; *P* =0.045), respectively. **Figure 3** illustrates the relationship of these metabolites to autoimmune hypothyroidism. However, after FDR adjustment, only indololactate was positively associated with autoimmune hypothyroidism (*P_FDR_* = 0.036). **Supplementary Table 15** displays the precise FDR outcomes, while **Table 2** shows the outcomes of the heterogeneity test and horizontal pleiotropic test. **Supplementary Table 18** presents detailed results of horizontal pleiotropy testing of gut metabolites and autoimmune hypothyroidism.

**Table 2:** Results of heterogeneity and horizontal pleiotropy in the MR analysis of gut metabolites for autoimmune hypothyroidism.

Gut metabolites	Heterogeneity test			Horizontal pleiotropy		
	Method	Q	Q_pval	Egger_ intercept	se	pval
Indolelactate	MR Egger	12.712	0.470	0.004	0.005	0.400
	IVW	13.924	0.455			
N- (3-furoyl)glycine	MR Egger	15.086	0.656	0.000	0.005	0.997
	IVW	15.086	0.717			
Pipecolate	MR Egger	23.663	0.086	-0.019	0.010	0.062
	IVW	23.962	0.077			
Alanine	MR Egger	42.915	0.075	0.008	0.007	0.238
	IVW	44.572	0.069			
Phenylalanine	MR Egger	3.799	0.284	-0.006	0.013	0.623
	IVW	4.106	0.392			
Allantoin	MR Egger	5.421	0.942	-0.013	0.007	0.070
	IVW	8.695	0.796			

### 3.3 Multivariable Mendelian randomization analysis

To determine the influence of the gut microbiota on the risk of autoimmune hypothyroidism, we further performed MVMR analysis. The consistent direction and magnitude of the different MR models further supported causal inference. It should be noted that both the family Defluviitaleaceae and genus DefluviitaleaceaeUCG011 were also suggestively significant in the MVMR (Family Defluviitaleaceae OR =0.158; 95% CI, 0.027-0.928; *P* =0.041; DefluviitaleaceaeUCG011 OR =0.164; 95% CI, 0.028-0.967; *P* =0.046) (**Table 3**), although failing to pass FDR correction. The MVMR results showed no independent relationship between gut metabolites and autoimmune hypothyroidism (**Table 4**).

**Table 3:** MVMR analysis results of gut microbiota and autoimmune hypothyroidism.

Gut microbiota	Methods	OR	CILower	CIUpper	P
Family.Defluviitaleaceae	IVW	0.158	0.027	0.928	0.041
Family.Defluviitaleaceae	Median	0.190	0.156	0.230	0.000
Family.Defluviitaleaceae	MR-Egger	0.138	0.024	0.802	0.027
Genus.DefluviitaleaceaeUCG011	IVW	0.164	0.028	0.967	0.046
Genus.DefluviitaleaceaeUCG011	Median	0.194	0.160	0.235	0.000
Genus.DefluviitaleaceaeUCG011	MR-Egger	0.149	0.026	0.868	0.034
Genus.Eggerthella	IVW	0.967	0.909	1.028	0.279
Genus.Eggerthella	Median	0.931	0.861	1.007	0.076
Genus.Eggerthella	MR-Egger	0.971	0.914	1.033	0.356
Genus.Intestinimonas	IVW	0.986	0.939	1.036	0.577
Genus.Intestinimonas	Median	0.964	0.899	1.033	0.298
Genus.Intestinimonas	MR-Egger	0.982	0.935	1.031	0.470
Genus.RuminococcaceaeUCG011	IVW	0.997	0.946	1.050	0.899
Genus.RuminococcaceaeUCG011	Median	1.025	0.958	1.097	0.478
Genus.RuminococcaceaeUCG011	MR-Egger	0.997	0.946	1.050	0.899
Genus.Subdoligranulum	IVW	0.979	0.911	1.052	0.569
Genus.Subdoligranulum	Median	0.974	0.882	1.077	0.614
Genus.Subdoligranulum	MR-Egger	0.984	0.916	1.058	0.664
Phylum.Actinobacteria	IVW	0.933	0.848	1.027	0.159
Phylum.Actinobacteria	Median	0.946	0.829	1.079	0.409
Phylum.Actinobacteria	MR-Egger	0.934	0.850	1.027	0.162

**Table 4:** MVMR analysis results of gut metabolites and autoimmune hypothyroidism.

Gut microbiota	Methods	OR	CILower	CIUpper	P
Indolelactate	IVW	1.019	0.824	1.259	0.862
Indolelactate	Median	1.039	0.831	1.297	0.741
Indolelactate	MR-Egger	1.040	0.833	1.297	0.732
N- (3-furoyl)glycine	IVW	0.997	0.977	1.018	0.813
N- (3-furoyl)glycine	Median	0.993	0.969	1.017	0.572
N- (3-furoyl)glycine	MR-Egger	0.996	0.974	1.017	0.696
Pipecolate	IVW	1.010	0.908	1.124	0.857
Pipecolate	Median	1.026	0.918	1.147	0.652
Pipecolate	MR-Egger	1.023	0.912	1.148	0.695
Alanine	IVW	0.962	0.621	1.490	0.862
Alanine	Median	0.739	0.452	1.209	0.229
Alanine	MR-Egger	1.644	0.330	8.199	0.544
Phenylalanine	IVW	0.792	0.591	1.061	0.118
Phenylalanine	Median	0.648	0.457	0.919	0.015
Phenylalanine	MR-Egger	0.791	0.589	1.063	0.120
Allantoin	IVW	1.005	0.869	1.163	0.942
Allantoin	Median	1.007	0.860	1.179	0.931
Allantoin	MR-Egger	0.988	0.847	1.154	0.882

### 3.4 Reverse MR analysis

In the reverse MR analysis, there was no evidence of a causal influence between autoimmune hypothyroidism and the identified gut microbiota (**Table 5**).

**Table 5:** Reverse causal association between autoimmune hypothyroidism and gut microbiota.

Exposure	Outcome	Methods	OR	CILower	CIUpper	P
Autoimmune hypothyroidism	Phylum.Actinobacteria	IVW	1.009	0.978	1.042	0.563
		Weighted median	1.017	0.968	1.069	0.507
		MR Egger	0.993	0.921	1.070	0.854
Autoimmune hypothyroidism	Genus.Intestinimonas	IVW	1.001	0.963	1.041	0.954
		Weighted median	0.995	0.938	1.056	0.878
		MR Egger	0.964	0.879	1.056	0.429
Autoimmune hypothyroidism	Genus.DefluviitaleaceaeUCG011	IVW	0.928	0.865	0.996	0.486
		Weighted median	0.993	0.881	1.120	0.911
		MR Egger	0.960	0.815	1.132	0.632
Autoimmune hypothyroidism	Genus.Eggerthella	IVW	1.025	0.964	1.089	0.428
		Weighted median	0.983	0.891	1.085	0.731
		MR Egger	0.932	0.809	1.074	0.334
Autoimmune hypothyroidism	Family. Defluviitaleaceae	IVW	0.990	0.943	1.039	0.692
		Weighted median	0.987	0.916	1.064	0.738
		MR Egger	1.022	0.912	1.145	0.692
Autoimmune hypothyroidism	Genus. Subdoligranulum	IVW	1.022	0.987	1.057	0.220
		Weighted median	1.008	0.954	1.066	0.773
		MR Egger	0.965	0.892	1.045	0.220
Autoimmune hypothyroidism	Genus. RuminococcaceaeUCG011	IVW	0.989	0.943	1.038	0.665
		Weighted median	0.991	0.917	1.070	0.812
		MR Egger	1.020	0.911	1.141	0.736

### 3.5 Enrichment analysis

Enrichment analysis of gut microbiota, metabolites and autoimmune hypothyroidism found that several key regulatory pathways were significantly enriched. GO enrichment analysis showed that 20 GO biological processes (e.g., transcription factor TFIID complex, collagen catabolic process, aminoacyltransferase activity, and disruption of plasma membrane integrity in another organism) were associated with autoimmune hypothyroidism (**Supplementary Figure 14**). KEGG enrichment analysis showed that the highest enrichments were Taurine and hypotaurine metabolism (*P*=5.17E-5), Transcriptional misregulation in cancer (*P*=9.00E-5), NOD-like receptor signaling pathway (*P*=2.00E-4), Calcium signaling pathway (*P*=6.64E-4), as well as MAPK signaling pathway (*P*=7.07E-4) (**Supplementary Figure 15**).

## 4 Discussion

### 4.1 Principal findings

This is the first MR analysis to examine the genetically predictive ability of gut microbiota and metabolites on autoimmune hypothyroidism. In the present MR study, we found borderline causal associations of phylum Actinobacteria, genus DefluviitaleaceaeUCG011, genus Eggerthella, family Defluviitaleaceae, genus Subdoligranulum and genus RuminococcaceaeUCG011 with a lower risk of hypothyroidism, while the genus Intestinimonas was borderline positively associated with autoimmune hypothyroidism. Additionally, in gut metabolites, indololactate was significantly positively associated with autoimmune hypothyroidism. The gut microbiota and its metabolites, also referred to as the thyroid-gut axis, may act directly or indirectly on the thyroid by influencing intestinal microelement uptake, iodothyronine conversion and storage, and immune regulation (29).

### 4.2 The association between the gut microbiota and host inflammatory regulation

The widely known fact is that a dysfunctional microbiota could adversely impact the immune system and host inflammatory regulation, potentially contributing to the development of autoimmune diseases such as autoimmune thyroid diseases (AITD) (30). HT, as the most common AITD worldwide, is characterized by chronic inflammation and autoantibodies against thyroid peroxidase (TPO) and thyroglobulin (TG). Previous research has demonstrated that the majority of HT cases eventually progress to autoimmune hypothyroidism (10).

### 4.3 The mechanisms of action for gut microbiota and gut metabolites

Moreover, gut dysbiosis could lead to alterations in the circulating levels of gut metabolites, which could exert an influence on the absorption and utilization of vital micronutrients for the thyroid gland (31). Short-chain fatty acids (SCFAs), being one of the primary components among these metabolites (32), are predominantly produced by the gut microbiota through the fermentation of dietary fibres. They held significant importance for the intestine. On the one hand, SCFAs serve as a major energy source for enterocytes (16). On the other hand, SCFAs, especially butyrate, exert a significant influence on immune regulation and possess anti-inflammatory properties (33, 34).

Notably, the uptake of iodine was primarily facilitated by the sodium/iodine symporter (NIS). Based on previous research, it was hypothesized that altering the release of SCFAs might be a potential mechanism through which the gut microbiota can influence the expression and activity of NIS and subsequently impact thyroid iodine metabolism (29). Furthermore, the microbiota could also influence the uptake and utilization of trace elements, such as iron and zinc. Particularly crucial was the fact that these trace elements played essential roles in regulating thyroid hormone synthesis and conversion: iron was indispensable for thyroid hormone synthesis, and zinc was needed for converting T4 to T3 (30).

The tryptophan transport system T was implicated in the counter transport of tri-iodothyronine and aromatic amino acids (35). Tryptophan is metabolized by the microbiota into indole derivatives, as well as tryptamine and kynurenine metabolites (36), which reduces the amount of tryptophan *in vivo*. Furthermore, our finding of indolelactate as a risk factor for autoimmune hypothyroidism was consistent with previous studies. Moreover, we identified N-(3-furoyl)glycine, pipecolate, phenylalanine and allantoin as microbial metabolites that served as protective factors against autoimmune hypothyroidism. These findings may offer novel insights for potential disease interventions.

### 4.4 Enrichment analysis

Furthermore, GO enrichment analysis found that larger number of GO biologic processes play key role in relationship between gut microbiota, metabolites and autoimmune hypothyroidism, which have been supported by previous studies. For example, collagen catabolic process was observed to be involved in the effects of gut microbiota/metabolites on autoimmune hypothyroidism, which had been proved that hypothyroidism leads to increased collagen-based stiffness (37). MAPK signaling pathway was also observed to be involved it, which had been demonstrated that the rat thyroid proliferation induced by TSH may involve an increase in MAPK signaling (38).

### 4.5 Strengths and limitations

One strength of our study was that we are the first to demonstrate causal associations among gut microbiota, metabolites and autoimmune hypothyroidism using univariate and multivariate MR, with various sensitivity analyses employed to ensure the consistency and robustness of our results. Second, the most recent extensive GWAS enables the acquisition and analysis of genetic data from sizable sample populations, thereby enhancing the robustness of findings in contrast to smaller randomized controlled studies. Finally, our examination goes a step further by refining the gut microbiota taxa and scrutinizing the causal impact of each taxon on autoimmune hypothyroidism from the genus to the phylum level. This establishes a theoretical foundation for understanding the subsequent mechanisms of specific bacterial strains on autoimmune hypothyroidism and aids in the identification of novel biomarkers. However, this study had several limitations. First, the sample size for autoimmune hypothyroidism was not very large. Second, although the majority of the data used in our study were European, a small number of the microbiological data were of other races, which may have confounded our estimates to some extent. Last, limited by the public database, we only verified the reverse causality between gut microbiota and autoimmune hypothyroidism and could not conduct a reverse causality study on gut metabolites. Further studies are certainly needed to validate our findings to provide more theoretical support for mechanistic research on the “thyroid–gut” axis.

## Data availability statement

Publicly available datasets were analyzed in this study. This data can be found in the article.

## Ethics statement

This study was approved by the Biomedical Research Ethics Committee of Shandong Provincial Hospital (SWYX2020-187).

## Author contributions

QG and HZ conceived the presented idea. XL and JY performed the computations and manuscript writing. SL, MT, XM, XW and YL were involved in the acquisition of data. YC, CK, QY, JL and LZ were involved in the interpretation of the data. All authors contributed to the article and approved the submitted version.
