# Supplementary material for: Investigating causal associations among gut microbiota, metabolites and autoimmune hypothyroidism: a univariable and multivariable Mendelian randomization study
Source: Front Immunol. 2024 Jan 4;14:1213159. doi: 10.3389/fimmu.2023.1213159 (PMC10794377; doi:10.3389/fimmu.2023.1213159)
Supplement: Supplementary file 1 [file DataSheet_1.docx]

Supplementary Material


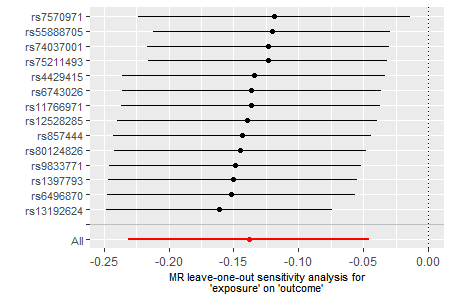


Supplementary Figure 1. Leave-one-out analysis of Phylum.Actinobacteria and autoimmune hypothyroidism.


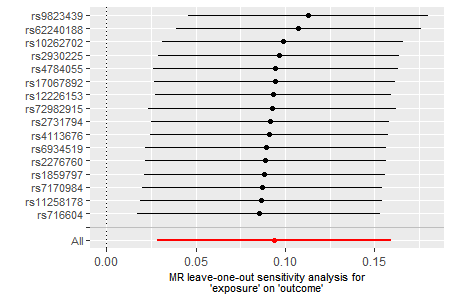


Supplementary Figure 2. Leave-one-out analysis of Genus.Intestinimonas and autoimmune hypothyroidism.


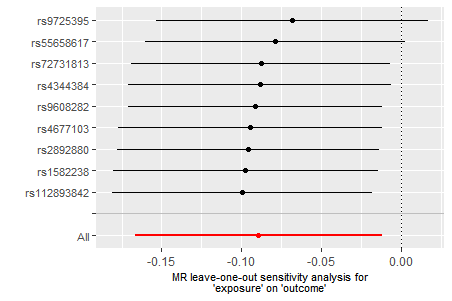


Supplementary Figure 3. Leave-one-out analysis of Genus.DefluviitaleaceaeUCG011 and autoimmune hypothyroidism.


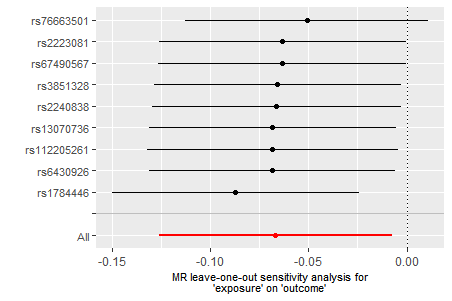


Supplementary Figure 4. Leave-one-out analysis of Genus.Eggerthella and autoimmune hypothyroidism.


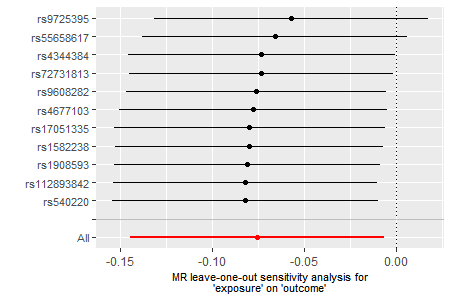


Supplementary Figure 5. Leave-one-out analysis of Family.Defluviitaleaceae and autoimmune hypothyroidism.


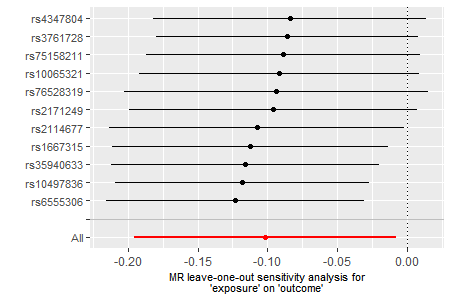


Supplementary Figure 6. Leave-one-out analysis of Genus.Subdoligranulum and autoimmune hypothyroidism.


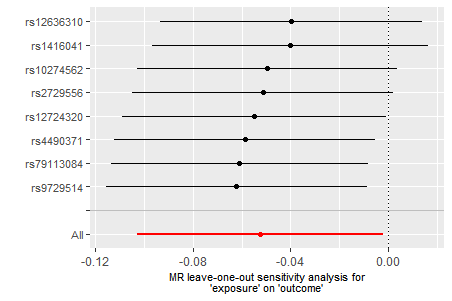


Supplementary Figure 7. Leave-one-out analysis of Genus.RuminococcaceaeUCG011 and autoimmune hypothyroidism.


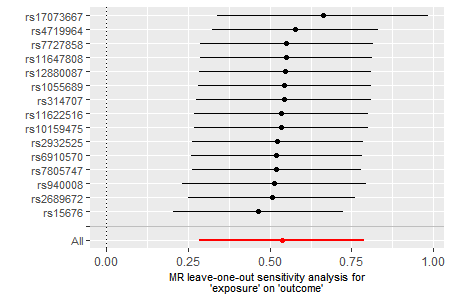


Supplementary Figure 8. Leave-one-out analysis of Indolelactate and autoimmune hypothyroidism.


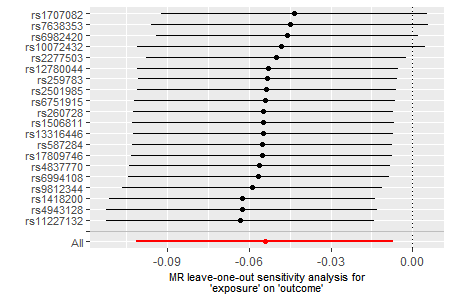


Supplementary Figure 9. Leave-one-out analysis of N- (3-furoyl)glycine and autoimmune hypothyroidism.


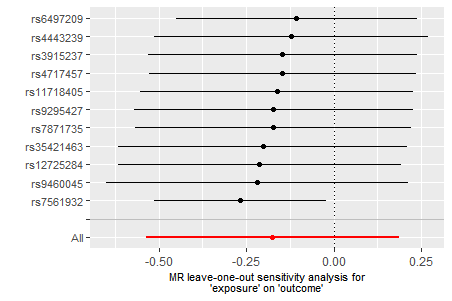


Supplementary Figure 10. Leave-one-out analysis of Pipecolate and autoimmune hypothyroidism.


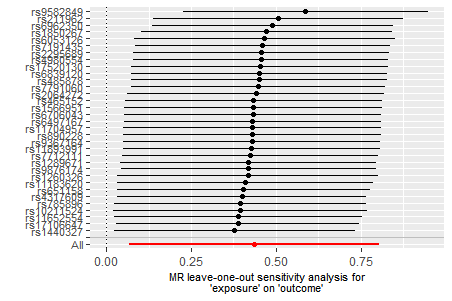


Supplementary Figure 11. Leave-one-out analysis of Alanine and autoimmune hypothyroidism.


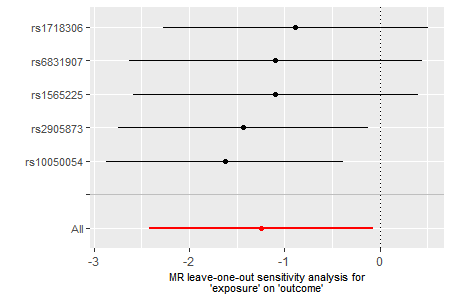


Supplementary Figure 12. Leave-one-out analysis of Phenylalanine and autoimmune hypothyroidism.


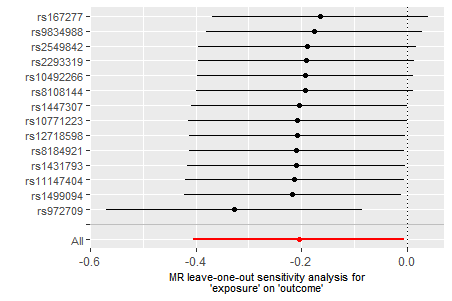


Supplementary Figure 13. Leave-one-out analysis of Allantoin and autoimmune hypothyroidism.


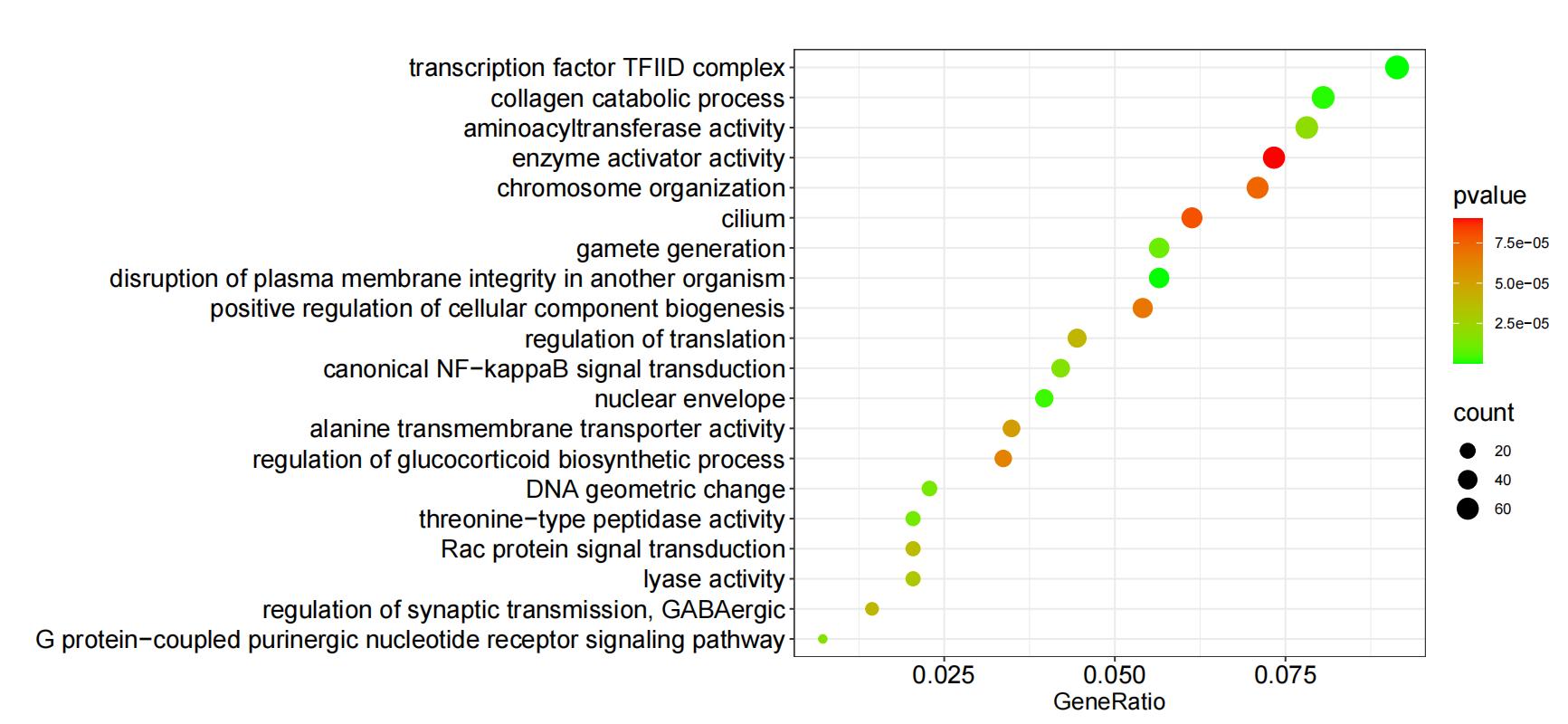


Supplementary Figure 14. Functional enrichment analysis of the network by using the gene ontology (GO).


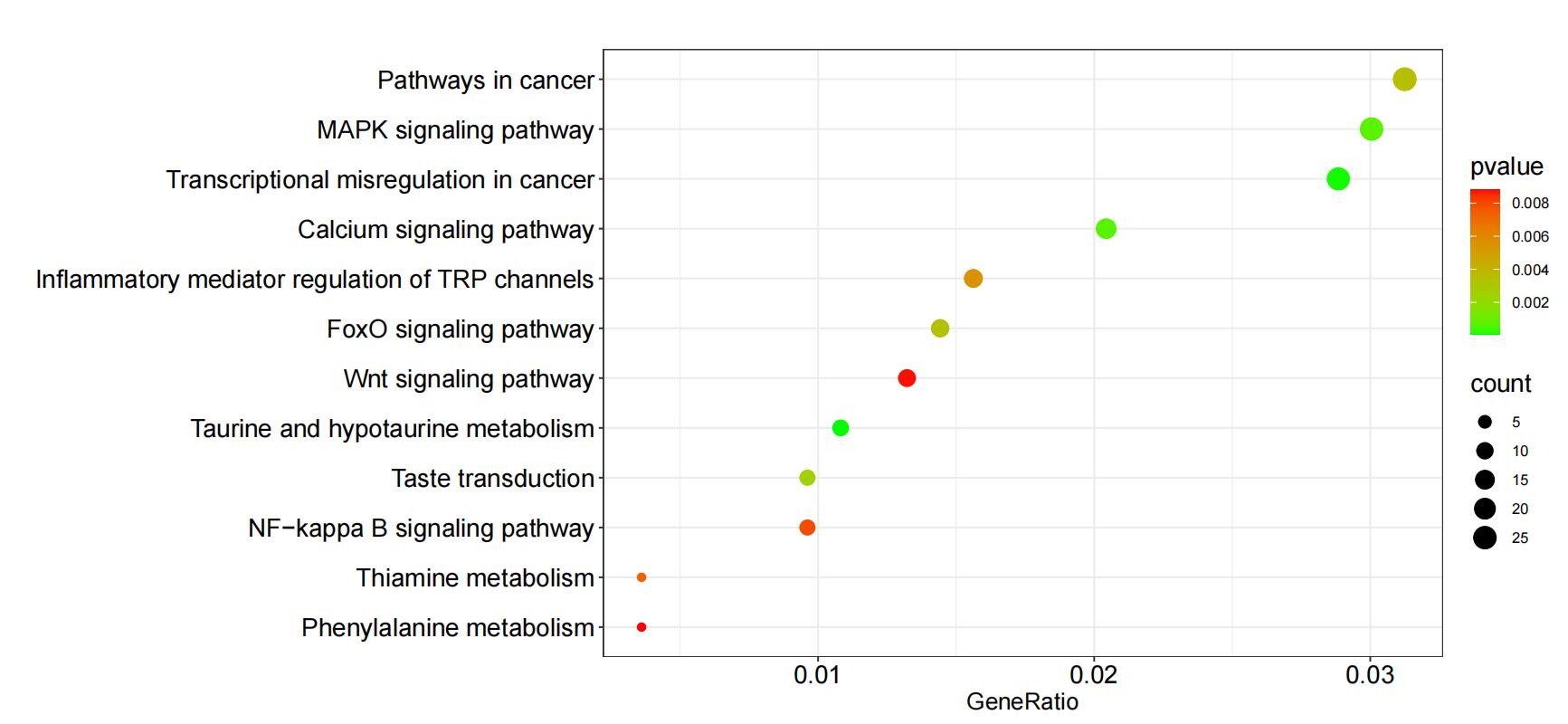


Supplementary Figure 15. Pathway enrichment analysis of the network by using the Kyoto Encyclopedia of Genes and Genomes (KEGG).
